# Congenital Acute Myeloid Leukemia with Unique Translocation t(11;19)(q23;p13.3)

**DOI:** 10.7759/cureus.289

**Published:** 2015-07-27

**Authors:** Chester K Yarbrough, S. Kathleen Bandt, Kyle Hurth, Jennifer A Wambach, Rakesh Rao, Shashikant Kulkarni, Francis V White, John L Frater, Jeffrey R Leonard

**Affiliations:** 1 School of Medicine, Washington University School of Medicine; 2 Neurological Surgery, Washington University School of Medicine; 3 Pathology, Neuropathology, Keck School of Medicine of USC; 4 Pediatrics, Newborn Medicine, Washington University School of Medicine; 5 Pathology and Immunology, Washington University School of Medicine; 6 Neurological Surgery, The Ohio State University, Nationwide Children's Hospital

**Keywords:** congenital leukemia, fetal leukemia, subdural hematoma, 11q23, mll rearrangement, 19p13.3

## Abstract

Congenital leukemia is rarely encountered in clinical practice, even in tertiary children's hospitals. Leukemia may cause significant coagulopathy, putting the patient at risk of intracranial hemorrhage. In this case, the authors present a female infant with a unique mixed phenotypic congenital acute myeloid leukemia showing mixed-lineage leukemia (MLL) rearrangement and severe coagulopathy resulting in a large subdural hematoma. Despite the fatal outcome in this case, neurosurgical treatment of patients with acute myeloid leukemia should be considered if coagulopathy and the clinical scenario allow.

## Introduction

Congenital leukemia, defined as leukemia diagnosed between birth and four weeks of life, is diagnosed in 0.2 to 4.7 in 1 million births [1-2], with acute myeloid leukemia (AML) more common than acute lymphoblastic leukemia (ALL) in the neonates [3-5]. In both congenital AML and ALL, the most common genetic defects, found in more than half of congenital leukemias, involve the rearrangement of the mixed-lineage leukemia (MLL) gene at chromosome 11q23 [6]. The prognosis remains poor [4, 7], with 23% and 26% survival in two large studies [3, 8]. Despite this, other authors have reported patients with long-term survival or even spontaneous remission with specific subtypes of acute leukemia [9–15]. Review of the literature revealed only one patient with congenital leukemia with intraparenchymal hemorrhage and fatal outcome [16]. The authors present their experience with a large subdural hematoma that likely occurred in utero secondary to congenital leukemia and profound coagulopathy.

## Case presentation

### History and physical examination

The female infant was born at 36 weeks estimated gestational age to a 28-year-old Gravida 1 mother whose pregnancy had been uncomplicated until 34 weeks gestation at which point she developed pre-term labor which subsequently resolved. The infant was delivered via Caesarean section for fetal decelerations following the spontaneous onset of labor. Apgar scores were 5 and 5 at 1 and 5 minutes of life, respectively. The infant was intubated shortly after birth for  respiratory distress. Initial examination of the skin was significant for a diffuse, non-blanching, bluish-red, macular rash with some palpable areas and multiple petechiae. The abdomen was full and somewhat distended with marked hepatosplenomegaly. The anterior fontanelle was slightly full and all cranial sutures were well-approximated. The pupils were minimally anisocoric (4 mm on the right, minimally reactive; 2 mm on the left, trace reactive). There was decreased spontaneous movement of the left upper and lower extremities with associated decreased tone throughout these extremities.

### Laboratory and pathologic evaluation

Initial laboratory studies demonstrated a white blood cell (WBC) count of 188,000 cells/cumm with 70% blasts, a hemoglobin of 9.2 g/dl, a platelet count of 110,000 cells/cumm, a partial thromboplastin time of 58.9 seconds, a prothrombin time of 65.1 seconds, and an international normalized ratio of 7.7. The patient showed laboratory findings concerning for tumor lysis syndrome, including elevated lactate dehydrogenase (> 76,000 IU/cumm; normal 130-700 IU/cumm), and uric acid (9.1 mg/dl; normal 2.0-6.0 mg/dl).

**Figure 1:**
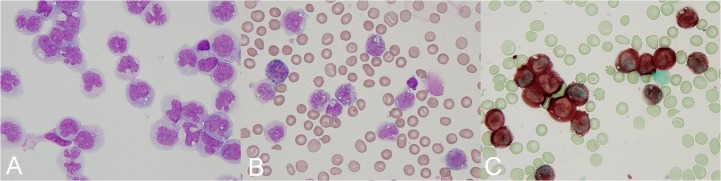
Peripheral blood smear and corresponding cytospin Preparation. Cytospin preparation (A) from the patient's peripheral blood smear shows the presence of malignant cells as described in the text. Cytochemical staining of the peripheral blood smear shows a subset of cells with MPO positivity (B) and strong diffuse positivity for NBE (C) [100x objective].

Examination of the peripheral blood smear and corresponding cytospin specimens showed blasts, the majority of which resembled monoblasts or promonocytes (Figure 1A). Cytochemical studies showed that a subset of blasts were positive for myeloperoxidase (MPO) and showed a strong diffuse positivity for alpha-naphthyl butyrate esterase (NBE) (Figure 1B, 1C). Flow cytometry showed the presence of an acute mixed phenotypic leukemia with 62% of events within the myeloid gate, and 19% of events within the monocytic gate. Cells falling within the myeloid gate showed coexpression of CD56, CD64, HLA-DR, TdT, and CD5 (partial). Cells falling within the monocytic gate showed coexpression of HLA-DR, CD13, CD14, CD56, and CD64. A pathologic diagnosis of AML with monocytic differentiation was given, and correlation with cytogenetic studies was recommended.

**Figure 2:**
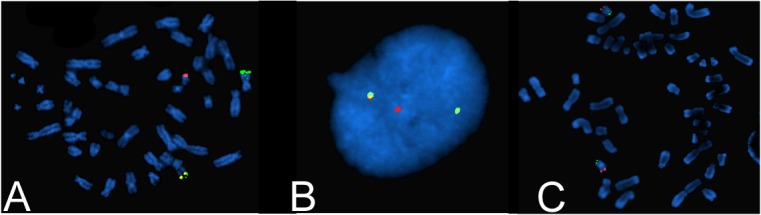
Flourescence in-situ hybridization reveals complex MLL rearrangement. A and B) 5' (green) and 3' (red) probes for MLL* *reveal a translocation of MLL* *with 5' portion on chromosome 11 and 3' on chromosome 19. C) Probes for chromosome 19 (green and red - p and q termini, respectively) reveal a more complex rearrangement or insertion has occurred.

Subsequent cytogenetic analysis demonstrated a karyotype of 46,XX,t(11;19)(q23;p13.3) in 20 of 20 analyzed metaphases. MLL gene rearrangement was confirmed by FISH, and there was no evidence of PML/RARA, AML1/ETO, or CBFB rearrangements (Figure 2A, 2B). No evidence of trisomy 8, trisomy 21, deletion/monosomy of 5q, or deletion/monosomy of 7q was detected. In addition, mitogen-stimulated lymphocyte culture showed no germline chromosomal abnormalities, consistent with chromosomal rearrangement. The observed translocation [t(11;19)(q23;p13.3)] was thought to be an MLL to ENL/MLLT-1 fusion. An additional chromosomal microarray analysis performed on peripheral blood was abnormal and showed a ~533Kb *de novo* interstitial deletion on the short arm of chromosome 19 (19p13.11) with a more complex translocation (Figure 2C). Placental pathology showed evidence of abnormal fetal myeloid cells, but no other histopathologic abnormality. Bone marrow biopsy was deferred due to severe coagulopathy.

### Imaging

**Figure 3:**
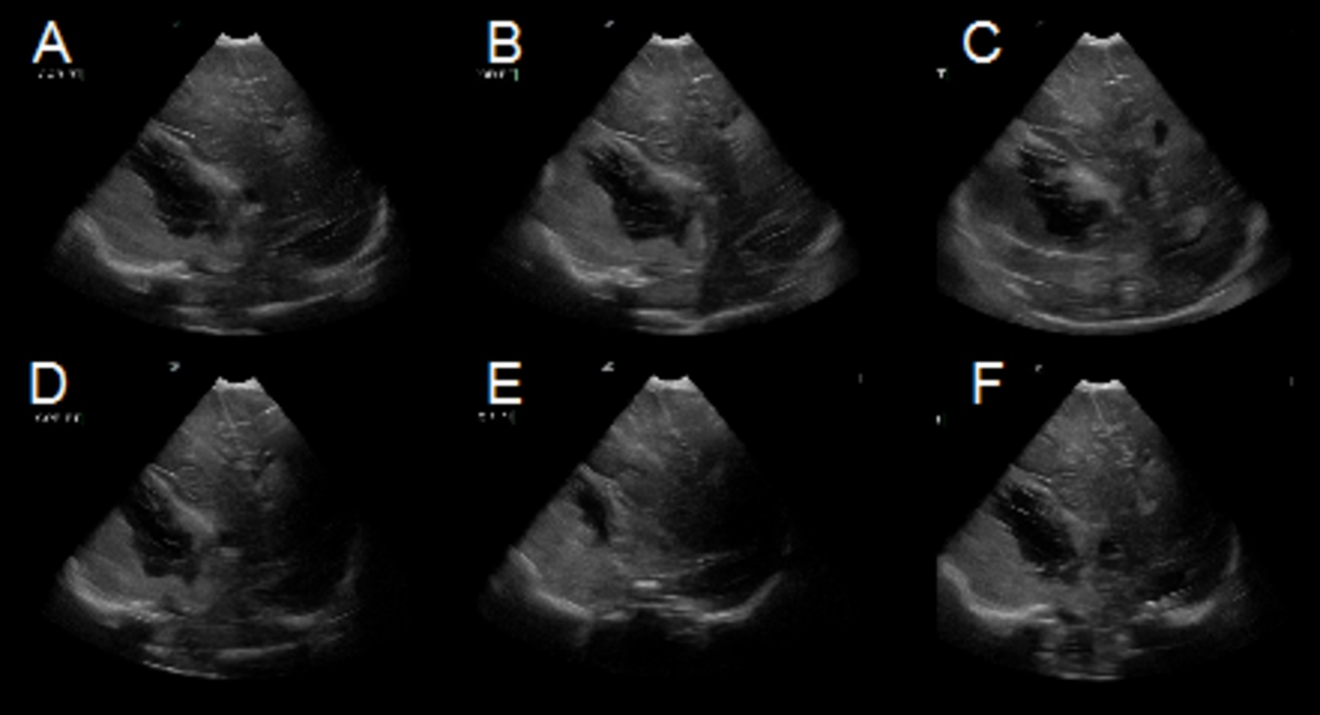
Coronal views of head ultrasound on Day of Life 2. Coronal views from posterior (A) to anterior (F) showing a large right-sided extra-axial hematoma.

**Figure 4:**
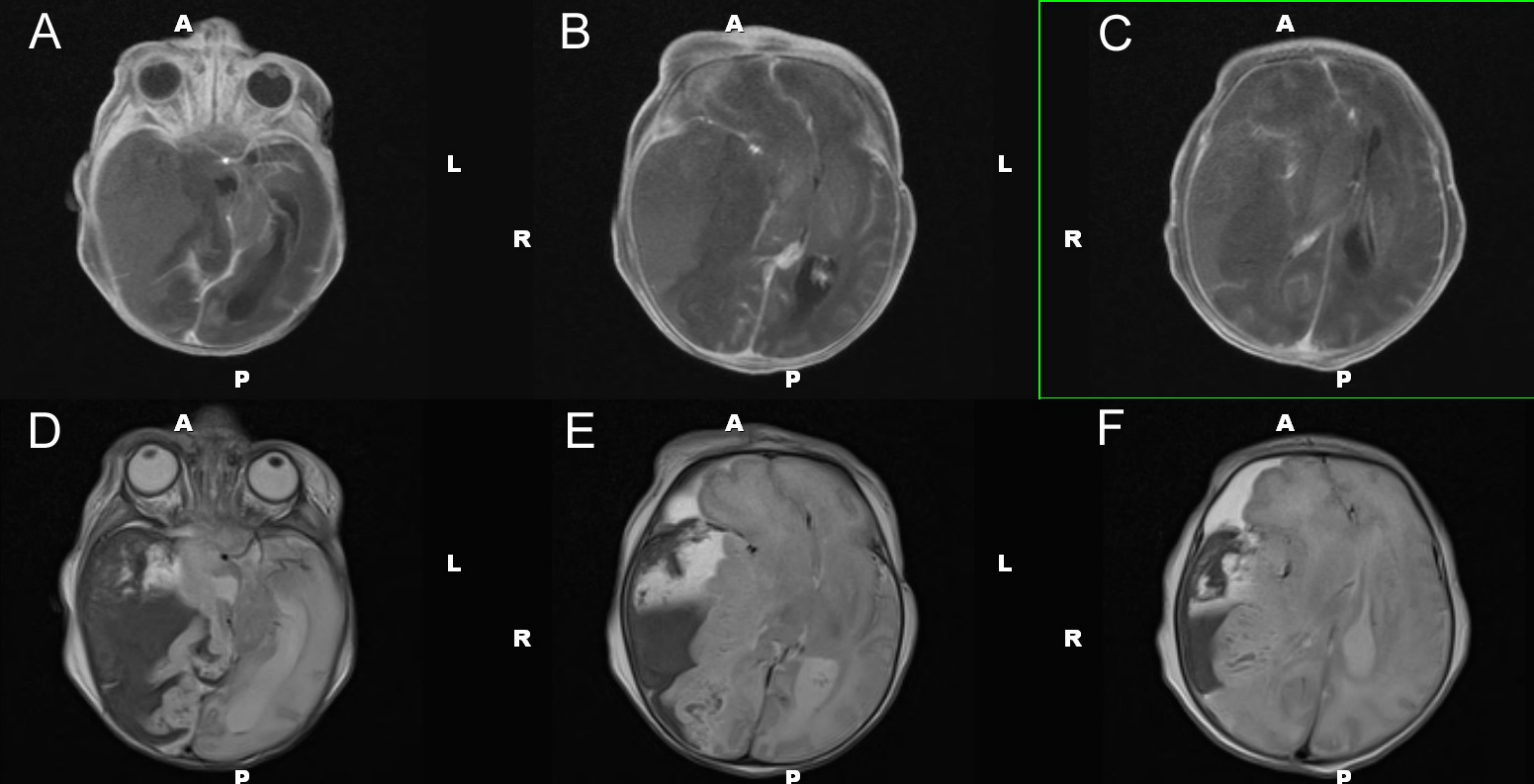
MRI of the brain - T1 with contrast and T2 axial views. T1 with contrast (A-C) and T2 (D-F) axial views characterizing the subdural hematoma.

**Figure 5:**
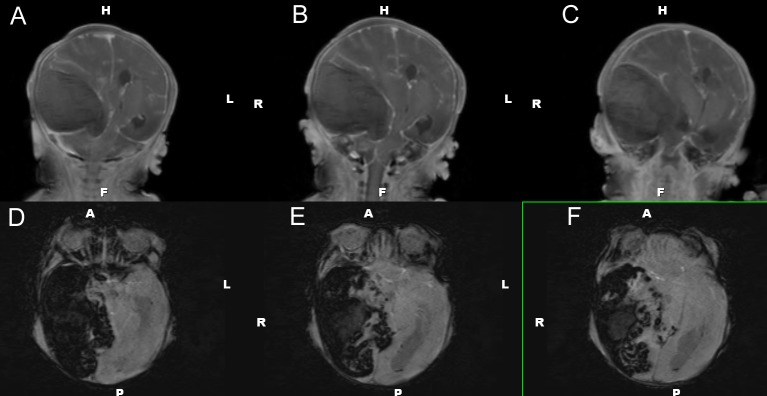
MRI of the brain – T1 coronal views and susceptibility-weighted imaging. T1 coronal (A-C) and susceptibility-weighted imaging (D-F) views characterizing the subdural hematoma.

**Figure 6:**
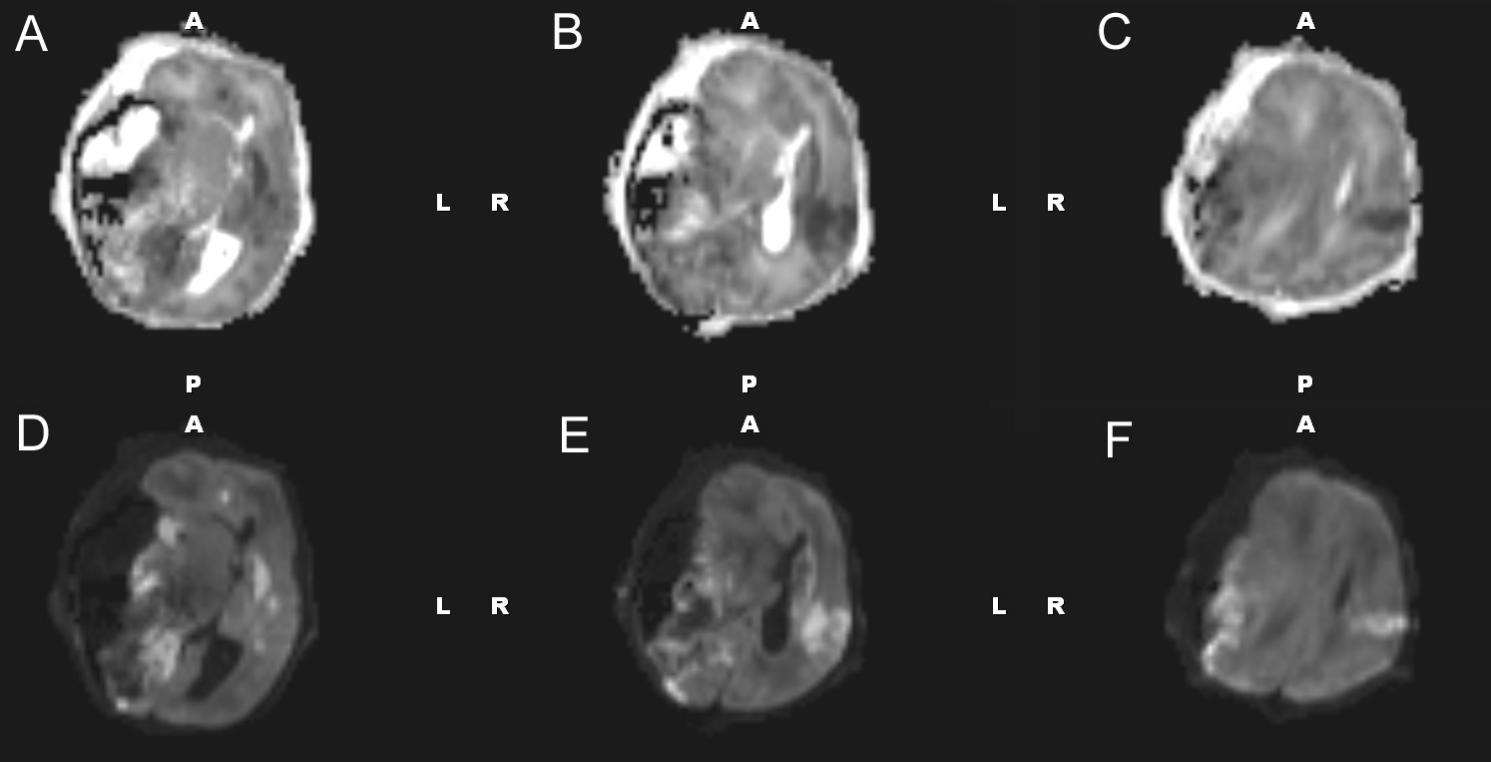
MRI of the brain – Diffusion and apparent diffusion coefficient imaging. Diffusion (A-C) and apparent diffusion coefficient (D-F) axial views characterizing the subdural hematoma.

Head ultrasound performed at 15 hours of life identified a 5.8 x 3.9 cm mixed density, echogenic lesion within the right middle cranial fossa (Figure 3). Ultrasound findings were consistent with an extra-axial fluid collection, likely subdural hematoma, but underlying chloroma versus vascular malformation could not be excluded. The ultrasound appearance suggested a large extra-axial hemorrhage of varying age, though exact dating was not possible. The patient subsequently underwent brain magnetic resonance imaging (MRI) at 21 hours of life for further characterization (Figures 4-6). MRI confirmed the nature of the large right middle cranial fossa lesion as a mixed intensity subdural hematoma with significant susceptibility artifact and identified smaller left temporal and right cerebellar subdural hematomas. T2 imaging revealed a fluid-fluid level with both hyper- and hypointense areas, suggestive of both acute and subacute hemorrhage (Figure 4). MRI also identified an uncal herniation with multifocal areas of diffusion restriction consistent with cerebral ischemia secondary to vascular compression and microvascular emboli. Entrapment of the left lateral ventricle was also noted. Diffuse enhancement of the leptomeninges was noted after contrast administration. 

### Treatment

The infant underwent serial exchange transfusions for management of hyperleukocytosis and received rasburicase® for tumor lysis syndrome. Once coagulation status permitted, the large right middle cranial fossa subdural hematoma was tapped through a patent right coronal suture. After a multidisciplinary discussion, including Neonatology, Neurosurgery, Hematology/Oncology and Neurology, the parents were counseled regarding the treatment options, including palliative neurosurgical interventions and aggressive chemotherapy in the setting of an overall grim prognosis. Ultimately, the patient’s family decided to redirect care toward comfort measures and the patient passed peacefully on Day of Life 4. 

### Post-mortem evaluation

**Figure 7:**
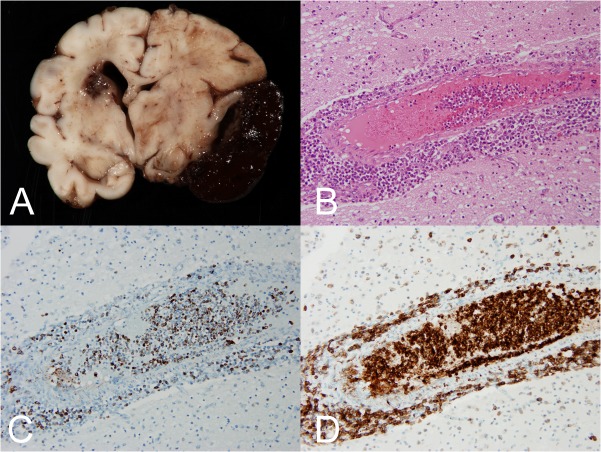
Post-mortem examination of the central nervous system. A) This coronal section reveals right-sided subdural hematoma, well-demarcated from the underlying parenchyma in most areas. Intraparenchymal hemorrhage is notable within the left caudate. Significant right-to-left midline shift is present. Photomicrograph demonstrating intravascular and perivascular involvement of the central nervous system by malignant cells using 20x objective [hematoxylin and eosin (B), MPO (C), and CD43 (D)].

Post-mortem evaluation confirmed the radiographic findings and showed the presence of a right middle cranial fossa hematoma involving not only the subdural space but also extending into the subarachnoid space and involving the parenchymal surface (Figure 7A). There was midline shift, compression of the right lateral ventricle, and mild expansion of the left lateral ventricle. Parenchyma adjacent to the hematoma was soft, dusky colored, and disrupted. Multifocal infarction was also present, particularly prominent in the head of the left caudate nucleus. In addition, gross findings of a transtentorial herniation, including anterior-posterior elongation of the midbrain and rostral pons, were present.

Histological examination of the central nervous system showed intravascular, perivascular, and leptomeningeal involvement by malignant-appearing mononuclear cells with hematopoietic features (Figure 7B). Mitoses were numerous (atypical mitoses were also identified), and many of the cells showed karyorrhexis. The cells showed strong myeloperoxidase (Figure 7C) and CD43 (Figure 7D) positivity, and were consistent with the diagnosis of AML established by hematopathological and cytogenetic analysis.

In addition to the CNS findings, the post-mortem examination showed the extensive involvement of multiple organ systems by AML blasts, many of which showed features of vascular/peri-vascular spread. Sections of rash-involved skin showed diffuse dermal involvement. Corresponding to the clinical finding of hepatosplenomegaly, the liver showed diffuse sinusoidal involvement by neoplastic cells with few remaining cords present and splenic red pulp was extensively involved and the spleen also showed extramedullary hematopoiesis. Neoplastic cells involved the pleural surfaces and the submucosa of the upper respiratory tract. The lungs showed neoplastic cells around bronchovascular bundles in the interstitium. There was full thickness involvement of the wall of the esophagus, stomach, and small and large intestines. In addition, blasts were present within sections of the: kidneys, heart, thymus, pancreas, spleen, pituitary, lymph nodes, adrenals, uterus, and ovaries. Bone marrow showed near 100% cellularity and was comprised predominantly of blasts.

The patient's placenta showed a maturation of chorionic villi consistent with the patient's estimated gestational age and was of an appropriate weight. The umbilical cord was three-vesseled, and fetal membranes were non-inflamed. There were no findings to suggest the presence of a TORCH agent. However, fetal blood showed the presence of immature myeloid cells, consistent with AML blasts.   

After the patient expired, verbal consent was obtained from the parents for public discussion of the patient's case. This case has been presented as a case review in NeoReviews as a neonatology case review, but the full cytogenetic and pathological details were not described in detail.

## Discussion

Congenital AML is a rare disorder that characteristically presents with leukemia cutis, an infiltration of neoplastic leukocytes and their precursors into the dermis (blueberry muffin rash), and hepatosplenomegaly [3]. Here, we present a case of a neonate with the classic findings of congenital AML, in addition to an abnormal neurologic examination and coagulopathy. A large middle fossa subdural hematoma caused severe compression of the diencephalon and brainstem, ultimately leading to the patient's death despite attempted drainage.

Diagnostic workup showed an AML with monocytic features and a unique translocation, t(11;19)(q23;p13.3), apparently resulting in fusion of MLL with ENL/MLLT-1. This translocation has been previously reported to occur in both congenital AML and ALL [17]. Studies examining the association between this specific translocation and leukemia have found generally poor survival with infants faring worse than older children and adults. However, patient outcomes vary by age and immunophenotype, and careful clinicopathological correlation is necessary for appropriate patient management [18].

The MLL gene serves as an upstream regulator of homeobox genes, and MLL gene rearrangements are believed to induce leukemia by altering normal homeobox gene expression in hematopoiesis [19-20]. The in-utero development of congenital leukemia has been shown in multiple studies, including concordant leukemia in twins, retrospective analysis of neonatal blood spots, examination of cord blood, and examination of fetal death [21-22].

Intervention on neonatal subdural hematoma may involve craniotomy, drain placement, percutaneous drainage, or subdural shunting procedure. In this case, only percutaneous drainage was felt to be safe after the appropriate hematologic correction had been instituted. Additional invasive procedures were discussed with the family but were not pursued. In one review of non-Down syndrome neonates with leukemia, the overall three-year survival rate was 26%, with a generally more favorable prognosis associated with AML versus ALL [4]. Thus, should clinical circumstances allow, neurosurgical intervention in this patient population is appropriate.

## Conclusions

Neonatal leukemia is a rare disorder with a poor, but not hopeless, prognosis. Hematologic abnormalities in this condition raise the risk of hemorrhage, which may benefit from surgical intervention. If surgical evacuation is attempted, surgeons should exercise caution when considering a craniotomy.

## References

[1] Bader JL, Miller RW (1979). US cancer incidence and mortality in the first year of life. Am J Dis Child.

[2] Pui CH, Kane JR, Crist WM (1995). Biology and treatment of infant leukemias. Leukemia.

[3] Bresters D, Reus AC, Veerman AJ, van Wering ER, van der Does-van den Berg A, Kaspers GJ (2002). Congenital leukaemia: the Dutch experience and review of the literature. Br J Haematol.

[4] Isaacs H Jr (1987). Congenital and neonatal malignant tumors. A 28-year experience at Children's Hospital of Los Angeles. Am J Pediatr Hematol Oncol.

[5] Sande JE, Arceci RJ, Lampkin BC (1999). Congenital and neonatal leukemia. Semin Perinatol.

[6] Eden T (2010). Aetiology of childhood leukaemia. Cancer Treat Rev.

[7] Kaneko Y, Shikano T, Maseki N, Sakurai M, Sakurai M, Takeda T, Hiyoshi Y, Mimaya J, Fujimoto Fujimoto (1988). Clinical characteristics of infant acute leukemia with or without 11q23 translocations. Leukemia.

[8] Isaacs H Jr (2003). Fetal and neonatal leukemia. J Pediatr Hematol Oncol.

[9] Classen CF, Behnisch W, Reinhardt D, Koenig M, Möller P, Debatin KM (2005). Spontaneous complete and sustained remission of a rearrangement CBP (16p13)-positive disseminated congenital myelosarcoma. Ann Hematol.

[10] Dinulos JG, Hawkins DS, Clark BS, Francis JS (1997). Spontaneous remission of congenital leukemia. J Pediatr.

[11] Grundy RG, Martinez A, Kempski H, Malone M, Atherton D (2000). Spontaneous remission of congenital leukemia: a case for conservative treatment. J Pediatr Hematol Oncol.

[12] Sainati L, Bolcato S, Cocito MG, Zanesco L, Basso G, Montaldi A, Piovesan AL (1996). Transient acute monoblastic leukemia with reciprocal (8;16)(p11;p13) translocation. Pediatr Hematol Oncol.

[13] Terui K, Sato T, Sasaki S, Kudo K, Kamio T, Ito E (2008). Two novel variants of MOZ-CBP fusion transcripts in spontaneously remitted infant leukemia with t(1;16;8)(p13;p13;p11), a new variant of t(8;16)(p11;p13). Haematologica.

[14] Wong KF, Yuen HL, Siu LL, Pang A, Kwong YL (2008). t(8;16)(p11;p13) predisposes to a transient but potentially recurring neonatal leukemia. Hum Pathol.

[15] Wu X, Sulavik D, Roulston D, Lim MS (2011). Spontaneous remission of congenital acute myeloid leukemia with t(8;16)(p11;13). Pediatr Blood Cancer.

[16] Ito M, Nishimaki S, Nakano Y, Tanaka F, Goto H, Yokota S (2009). A case of fetal leukemia with intracranial hemorrhage and early-onset jaundice. Fetal leukemia with intracranial hemorrhage and jaundice. Arch Gynecol Obstet.

[17] Chessells JM, Harrison CJ, Kempski H, Webb DK, Wheatley K, Hann IM, Stevens RF, Harrison G, Gibson BE, MRC Childhood Leukaemia working party (2002). Clinical features, cytogenetics and outcome in acute lymphoblastic and myeloid leukaemia of infancy: report from the MRC Childhood Leukaemia working party. Leukemia.

[18] Rubnitz JE, Camitta BM, Mahmoud H, Raimondi SC, Carroll AJ, Borowitz MJ, Shuster JJ, Link MP, Pullen DJ, Downing JR, Behm FG, Pui CH (1999). Childhood acute lymphoblastic leukemia with the MLL-ENL fusion and t(11;19)(q23;p13.3) translocation. J Clin Oncol.

[19] Eguchi M, Eguchi-Ishimae M, Greaves M (2005). Molecular pathogenesis of MLL-associated leukemias. Hematol.

[20] Hayashi Y (2000). The molecular genetics of recurring chromosome abnormalities in acute myeloid leukemia. Semin Hematol.

[21] Greaves M (2005). In utero origins of childhood leukaemia. Early Hum Dev.

[22] Hunger SP, McGavran L, Meltesen L, Parker NB, Kassenbrock CK, Bitter MA (1998). Oncogenesis in utero: fetal death due to acute myelogenous leukaemia with an MLL translocation. Br J Haematol.

